# Editorial: COVID-19 and vaccination-associated ocular complications

**DOI:** 10.3389/fcimb.2024.1413435

**Published:** 2024-05-06

**Authors:** Xinyuan Zhang, Rachael Niederer, Kelvin Yi Chong Teo

**Affiliations:** ^1^ Beijing Tongren Eye Center, Beijing Tongren Hospital, Capital Medical University, Beijing, China; ^2^ Beijing Retinal and Choroidal Vascular Diseases Study Group, Beijing Tongren Hospital, Beijing, China; ^3^ Department of Ophthalmology, University of Auckland, Auckland, New Zealand; ^4^ Singapore Eye Research Institute, Singapore National Eye Centre, Singapore, Singapore

**Keywords:** COVID-19, SARS-CoV-2 virus, vaccination, ocular disorders, sequelae

COVID-19, caused by the SARS-Cov-2 virus, has swiftly spread globally since 2019. According to the World Health Organization, by March 10, 2024, more than 775 million people had contracted SARS-CoV-2 and over 7 million deaths had occurred secondary to the virus. The severity of COVID-19 can range from asymptomatic or mild to severe respiratory distress and death. Severe cases of COVID-19 lead to pneumonia, acute respiratory distress syndrome, and respiratory failure. SARS-Cov-2 virus can also affect other organs, including cardiovascular complications, neurological sequelae, gastrointestinal complications, and dermatologic manifestations.

Increasing evidence has raised concerns about the associated ocular disorders in patients with SARS-CoV-2 infection or COVID-19 vaccination. The virus can affect the eyes directly or indirectly. The reported ocular manifestations due to COVID-19 vary greatly and include dry eye, conjunctivitis, scleritis, uveitis, endophthalmitis, retinopathy and retinal vascular occlusions, acute glaucoma, optic neuropathy, and cranial nerve palsies. In a questionnaire survey study, Ma et al. reported that the degree of ocular inflammatory activity within 3 months before SARS-CoV-2 infection is a major recurrence risk factor. To investigate if ocular tissue and potential infectivity of lacrimal fluid ocular infection of SARS-Cov-2, Kimpel et al. found that SARS-Cov-2 could be detected in the eyes of some of the infected patients, albeit at lower levels compared to nasopharyngeal swabs. They also found that the lacrimal fluid can be infectious with high viral load in these patients. The impact of SARS-CoV-2 on periocular injection pain and hypersensitive reactions is a specific and somewhat niche area of interest within the broader scope of COVID-19-related research. While comprehensive studies addressing this topic might be limited, we can infer potential impacts based on the known effects of COVID-19 on the body’s immune response and the potential for increased sensitivity or inflammatory reactions. In an interesting study, Liu et al. investigated various indicators in patients who had revived botulinum toxin A injections at the same site before and after their infection, including pain scores, allergic reactions, and the occurrence of complications, suggesting administering botulinum toxin type A three weeks after COVID-19 recovery is a justifiable and safe approach.

While COVID-19 vaccines are generally safe and effective in preventing severe illness, hospitalizations, and deaths due to the virus, there have been rare reports of ocular disorders following vaccination, such as uveitis, adenoviral conjunctivitis, retinal vascular occlusions, optic neuritis, and possibly corneal transplant rejection. Tajunisah et al. reported cases of ocular complications after vaccination, including the Pfizer-BioNTech vaccine and Oxford AstraZeneca, in three different eye centers in Malaysia. They reported four cases with acute retinal pigment epitheliitis (within 3h of vaccination), unilateral choroidal neovascularization (3 days after vaccination), bilateral acute multifocal placoid pigment epitheliopathy (a week after vaccination) as well as herpes zoster infection, and unilateral anterior non-granulomatous uveitis (2 weeks after vaccination). The possible mechanism remains unclear, they postulated that a possibility of the vaccine-mediated immune response leads to an increase in the inflammatory mediators. In a review article, Allen et al. provided a comprehensive review of the perceived correlation between COVID-19 vaccination and corneal allograft rejection by reviewing 262 articles. They concluded that corneal allograft rejection appears to be a rare complication of COVID-19 vaccination most frequently observed in high-risk corneal transplants. In a mini review, Shi and Danesh-Meyer reported that optic neuritis is the most commonly occurring neuro-ophthalmic sequelae following COVID-19 vaccination or infection.

As the pandemic progresses, marked by the emergence of new viral variants and the concurrent development of vaccines to meet these challenges, it becomes imperative to thoroughly understand and appreciate the multifaceted potential consequences. Additionally, since the eyes can reveal signs of various systemic diseases, a comprehensive understanding of ocular complications associated with COVID-19 can enhance the diagnosis and treatment of the systemic manifestations of COVID-19 and its aftereffects. Furthermore, the underlying mechanisms of pathogenesis of many of these sequelae remain poorly understood; animal, preclinical studies, and clinical trials will be warranted to provide more pathophysiological information on COVID-19 and vaccination-associated ocular disorders.

In summary, this Research Topic has covered survey studies, case reports, and case-controlled studies, and has explored the broad range of ocular sequelae and highlighted the latest advancements in scientific and clinical principles regarding the diagnosis, progression, and management of individuals with ocular disorders associated with COVID-19 or vaccination. In this rapidly evolving field, collaboration between the scientific community and clinicians is indispensable to unravelling the mysteries and gaining a deeper understanding of the complexities surrounding COVID-19 infection and the ocular sequelae of vaccination.

## Author contributions

XZ: Writing – original draft, Writing – review & editing. RN: Writing – review & editing. KT: Writing – review & editing.

